# First person – Ruth Howe

**DOI:** 10.1242/dmm.053191

**Published:** 2026-09-01

## Abstract

First Person is a series of interviews with the first authors of a selection of papers published in Disease Models & Mechanisms, helping researchers promote themselves alongside their papers. Ruth Howe is first author on ‘Expanding the scope of *Mycobacterium abscessus* reference strains to improve pulmonary disease modelling’, published in DMM. Ruth is a Clinical and Research Fellow in the lab of Gyanu Lamichhane at Johns Hopkins University, Baltimore, MD, USA, investigating models and new therapeutic modalities for nontuberculous mycobacterial lung diseases.

**Figure:**
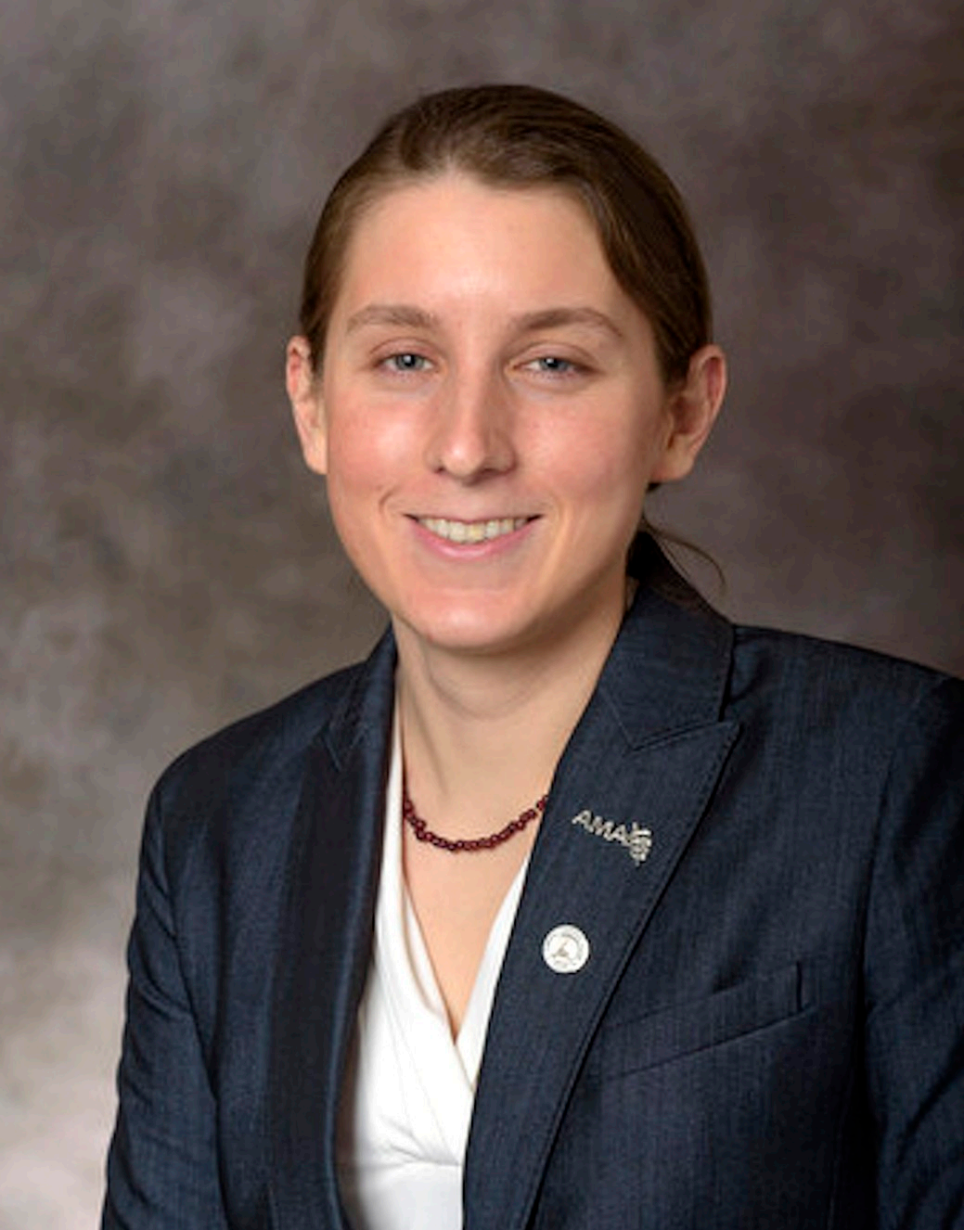
Ruth Howe

**Who or what inspired you to become a scientist?**

My brother and I had a free-range childhood in the Appalachian mountains, and I would credit the unsupervised, screen-free years spent hunting salamanders and climbing trees to watch the moss grow or spy on deer foraging with inspiring the love and curiosity that has ultimately led me into science. However, what nudged me into this career rather than, say, a fulfilling life of nature writing was having several early mentors who stood up for me as a girl interested in extremely technical things (so taboo!) and later mentors who encouraged me to keep going and helped me navigate the training landscape. This started in middle school, when my friend's father who worked at the university, Mr Locascio, was excited that someone was interested in centrifuges and helped me find the equations I needed to build my own with my dad out of an old washing machine with new wooden gearing. After that, another friend's father, Dr Dickerman, and his technician, Elena Shulaeva, took me under their wings to teach me basic molecular biology lab techniques and strongly encouraged me to switch my college plan from architecture to biology. When my high-school principal called me and my co-valedictorian to his office and told us our dreams were impossible, these mentors and our parents came down hard in our defence. Again, after university, my PI Dr Odom-John sat me down and gave me a pep talk to go try for a dual MD and PhD so that even more research doors would open. Each of these people had infectious enthusiasm for their fields and clearly wanted to share them with the next generation. All of their many mentees are extremely lucky to share in that joy. If that kind of perpetual wonder at exploring the world was typical of a scientist, how could I not want to be one myself?

**What is the main question or challenge in disease biology you are addressing in this paper? How did you go about investigating your question or challenge?**

*Mycobacterium abscessus* lung disease is caused by a diverse set of subspecies with differing drug resistance capabilities determined at both the genetic and morphologic level. Investigating basic biology of these organisms and developing new treatments has been hampered by widespread availability of just one *M. abscessus* reference strain, which was never known to cause lung disease (it was originally isolated from a knee abscess) and which we suspected was no longer adequate to address the complex questions being posed by this growing field of research. We set out to compare this reference strain, ATCC 19977, against 15 *M. abscessus* isolates taken from patient lungs in two different geographic settings that represented both of the major clinically encountered subspecies and both bacterial morphologies, all of which are thought to be contributing factors to disease development. We first characterized *in vitro* growth habits and drug susceptibilities for all strains, then characterized their growth habits and resulting disease pathology in the lungs of mice. Comparing these *in vitro* and *in vivo* profiles for each patient-derived *M. abscessus* strain to ATCC 19977 allowed us to determine that ATCC 19977 behaves differently from the *M. abscessus* we see in patients in ways that impact what kinds of clinical questions we can use it to answer. Although we expect that this growing field will need even more models in the future tailored to future needs of the field, we identified a set of five patient-derived *M. abscessus* strains that more closely aligns with the questions we want to ask next in our own work and which we think would facilitate the work of our colleagues today.

**How would you explain the main findings of your paper to non-scientific family and friends?**

Any job is easier if you use the correct tools. While you might be able to pound in a nail with the back of a screwdriver and some effort, you don't want to try rebuilding a deck with a spork, two rolls of kitchen towels, a jar of honey and no plan. Something similar is true for science. When we want to treat a disease, we need the right tools to help us figure out how the disease works and then test new treatments to see which is best. For the disease our lab works on – a dangerous bacteria called *M. abscessus* – we didn't have the right tools to figure out how to treat it. At best, we were using a screwdriver instead of a hammer to pound in our nails, so it was taking a lot of effort to do simple things. For some of our more complicated questions, though, we were trying to build a deck with sporks and honey and couldn't figure out how to go about it at all. The work we describe in this publication is essentially testing a bunch of potential tools so that our workbench now has a hammer, a power drill and a saw that we can use for future projects rather than relying on the spork and the screwdriver.

With the additional models we present here, we hope that we can help researchers ask questions specific to particular *M. abscessus* morphotypes or subspecies, decrease the animal numbers and expenses needed for modelling, and expedite both drug development and basic biological research into *M. abscessus*

**What are the potential implications of these results for disease biology and the possible impact on patients?**

Previously, it has been expensive, difficult and unreliable to model pulmonary *M. abscessus* disease for drug development. What questions the field could ask were limited by access to just a single, lab-adapted reference strain when the subspecies and morphologic diversity of the human pathogen is expansive. With the additional models we present here, we hope that we can help researchers ask questions specific to particular *M. abscessus* morphotypes or subspecies, decrease the animal numbers and expenses needed for modelling, and expedite both drug development and basic biological research into *M. abscessus*. That will get new medications to patients faster and with lower systemic costs than we could previously support.

**Figure:**
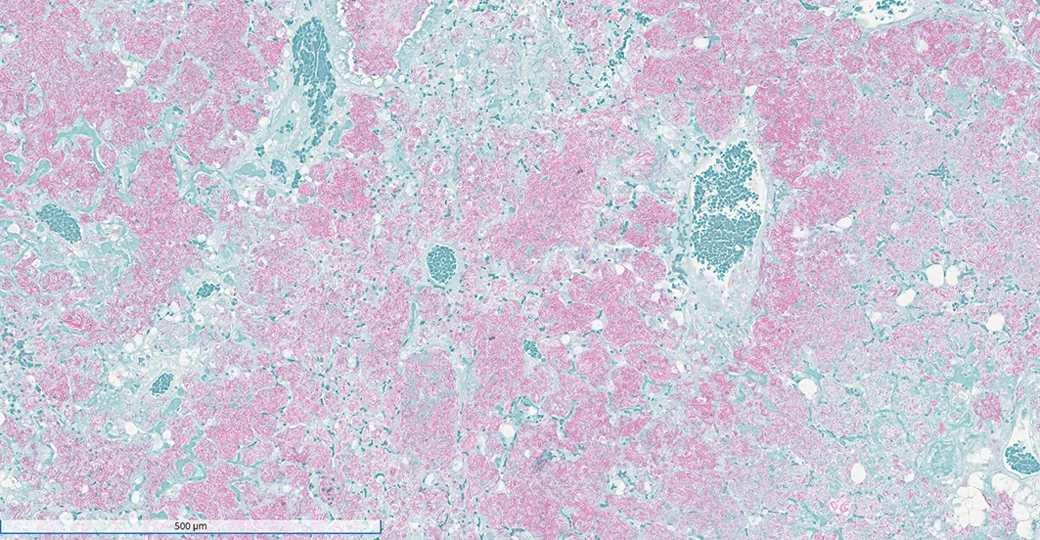
*Mycobacterium abscessus* (in pink) invading the lungs of C3HeB/FeJ mice.

**Why did you choose DMM for your paper?**

This work was all about bringing better tools to the table for ourselves and our colleagues in a field that is still finding its feet and beginning to be constrained by the lack of adequate models for the questions we want to ask. We wanted to put our findings exactly where people would look for them: in a journal about disease models.

**Given your current role, what challenges do you face and what changes could improve the professional lives of other scientists in this role?**

Being a dual laboratory scientist and clinician can be a fine balancing act in terms of time commitments necessary to stay current in both domains as well as the tremendous obligation of our social contract to be engaged in public scientific discourse and advocacy on behalf of both our patients and science. It is also an extremely long training pathway with generally minimal financial compensation, all of which discourages and eliminates some of our finest minds early in the process. As a scientific community and as a broader society, we suffer for that attrition. Having come through the Medical Scientist Training Program (MSTP) and American Board of Internal Medicine (ABIM) Research Track after a few years of working in the ‘real world’ and watching my spectacular colleagues take their own paths, I do think that there are some fairly straightforward measures we could take to attract and retain brilliant talent. Financial compensation commensurate to overall level of training is critical for a training path that requires multiple backtracks in terms of salary grade and can delay entry into faculty or industry positions until one's late 30s or early 40s. That kind of adaptive compensation might help us attract scientists, engineers, programmers and clinicians looking to make a mid-career switch and enrich our field, but who can't commit to living below the poverty line for four or more years of cross-training. We would also benefit from acknowledging public engagement as an essential part of a physician–scientist role and offering protected, paid time for those efforts.

**What's next for you?**

Science! Our lab has several wild projects up our sleeves that will be coming to publication over the next year or so, which I am excited to share before I complete fellowship in July 2027. After fellowship, I am open to staying in academia if feasible, since I entered this field with the specific aim of public service through discovery. However, the current research environment requires flexibility, and there can be substantial power in public–private partnerships. If I can better serve my patients by moving away from clinical practice and entering a full time industry research position, it would be both an honour and a great adventure to do so.

**Tell us something interesting about yourself that wouldn't be on your CV**

My path to science certainly wasn't straightforward and that is reflected on my CV; it took a lot of odd jobs to pay my way through college and after, and I admit I didn't always keep food on the table. Some exploits that didn't make it to the CV would be the musical ones. I've played Western classical, Celtic, Mariachi and jazz violin since I was a kid, though I haven't been in a band since COVID. Our mariachi band used to get paid in kitchen leftovers after the gig, and, on one memorable occasion, we got paid in boots.
